# Current practice of lung ultrasonography (LUS) in the diagnosis of pneumothorax: a survey of physician sonographers in Germany

**DOI:** 10.1186/s13089-014-0016-y

**Published:** 2014-10-15

**Authors:** Thomas Berlet, Tobias Fehr, Tobias M Merz

**Affiliations:** 1 Department of Intensive Care Medicine, Inselspital/Bern University Hospital and University of Bern, Bern, Switzerland

## Abstract

**Background:**

The purpose of this study was to survey the current practice of the use of lung ultrasonography (LUS) in the diagnosis of pneumothorax.

**Methods:**

Physician sonographers, accredited for diagnostic ultrasonography in surgery, anaesthesia and medicine were studied. Questions addressed the frequency of exposure to patients with suspected pneumothorax, frequency of LUS use, preferences regarding technical aspects of LUS examination, assessment of diagnostic accuracy of LUS and involvement in teaching.

**Results:**

Of the respondents, 55.1% used LUS ‘always’ or ‘frequently’ for suspected pneumothorax. Also, 35.5% of physicians rated LUS as ‘always reliable’ in ruling out pneumothorax, and 21.3% of respondents rated LUS as ‘always reliable’ in ruling in pneumothorax. The mode of performing LUS for pneumothorax was highly variable.

Statistically significant differences were found regarding the likelihood of LUS usage, the combined use of M-Mode and B-mode scanning and the confidence to exclude pneumothorax based on LUS findings for physicians with frequent exposure to pneumothorax cases.

**Conclusions:**

Physicians' use of LUS in the diagnosis of pneumothorax is modest. Confidence in diagnostic accuracy is not comprehensive. Further research is required to establish the most efficient way of performing LUS in this scenario to achieve the highest possible diagnostic accuracy and reliable documentation of examination results.

## Background

Pneumothorax is a frequent problem in various medical fields, such as emergency medicine, respiratory care, surgery, interventional radiology, critical care and anaesthesia [1].

Lung ultrasonography (LUS) is well suited for the diagnosis of pneumothorax and yields better results than conventional chest X-ray [2]. Despite the proven efficacy of point-of-care sonography in the management of patients with suspected pneumothorax in selected clinical settings, controversy regarding its diagnostic accuracy continues [2],[3]. In addition, there appears to be a narrow knowledge base regarding technical aspects such as transducer selection, transducer position and scanning mode as well as the most appropriate mode of reproducible documentation of the findings of LUS. Recommendations are either vague or contradictory [2],[4].

In view of the scarce evidence regarding the best way of applying LUS in the setting of suspected pneumothorax and the on-going controversy over its diagnostic accuracy, the study at hand was designed to assess the reality of LUS usage in a large number of expert medical sonographers. By approaching members of various sections and working groups of the German Society for Ultrasound in Medicine (DEGUM), we expected to benefit from the experience of physicians practising in different areas of clinical medicine in which patients with pneumothorax are encountered.

The aim of this study was to determine (a) to what extent ultrasonography is used by physicians involved in the management of patients with suspected pneumothorax, (b) which transducers, transducer orientations and ultrasound modes are preferred, (c) how images are stored and results documented, (d) what degree of diagnostic accuracy experienced users attribute to LUS for diagnosis of pneumothorax, and finally, if (e) physicians are actively involved in training colleagues and staff in the use of LUS.

## Methods

A questionnaire, comprising ten questions was developed. Questions pertained to frequency of involvement in the management of patients with suspected pneumothorax, frequency of LUS use in this setting, preferences regarding technical aspects of LUS use, physicians' perception of diagnostic accuracy and involvement in teaching of LUS (Additional file 1). Physician contact details were obtained from a publicly available directory of physician sonographers accredited by the German Society for Ultrasound in Medicine (DEGUM) in the use of ultrasonography in surgery, anaesthesia, general medicine, emergency medicine or chest medicine [5]. Accreditation requires completion of both specialist training and formal ultrasound training. Accredited physician sonographers are self-reliant in performing and reporting diagnostic ultrasound examinations within their area of expertise. In total, 337 physicians were contacted either by e-mail (*n* = 293) or by post (*n* = 44). The purpose of the study was explained, and physicians were invited to complete the survey on-line by using the SurveyMonkey® web site (https://de.surveymonkey.com/) or to return a printed copy of the survey by post. A reminder was sent to all physicians 3 weeks after the initial mailing. The survey was conducted over a 6-week period starting in April 2013. Responses to the survey were anonymised.

### Statistical methods

Categorical data are presented as counts per group or percentages. Statistical analysis was performed using StatView® software (Abacus concepts, Berkeley, CA, USA). Fisher's exact test was used for comparison of categorical data. Datasets with missing values were included in analysis; missing values were not replaced. All tests of statistical significance were two-sided. A *P* value <0.05 was considered statistically significant.

## Results

Responses were received from 89 accredited physician sonographers. The electronic version of the survey was used by 77 respondents.

Seventy-seven respondents (86.5%) were directly involved in the management of patients with suspected pneumothorax up to several times per month but less frequently than several times per week. Forty-nine respondents (55.1%) used LUS either ‘always’ or ‘frequently’ when managing cases of suspected pneumothorax. Correlation between exposure to cases of suspected pneumothorax and the likelihood of LUS usage is illustrated in Table 1. Physicians who were frequently or very frequently involved in the management of pneumothorax cases used LUS in a higher proportion of cases; the opposite held true for physicians managing suspected pneumothorax cases infrequently (*p* = 0.015).

**Table 1:** Involvement frequency in management of suspected pneumothorax and lung ultrasonography (LUS) usage in patients with suspected pneumothorax

**LUS-usage**	**Frequency of involvement**			
	**Infrequently**	**Frequently**	**Very frequently**	**Total**
	**≤1/month**	**≤1/week**	**>1/week**	
Never/rarely	7.9%	9.0%	1.1%	18.0%
	(7)	(8)	(1)	(16)
Occasionally	14.6%	11.2%	1.1%	27.0%
	(13)	(10)	(1)	(24)
Frequently	4.5%	11.2%*	1.1%*	16.9%
	(4)	(10)	(1)	(15)
Always/almost always	5.6%	22.5%*	10.1%*	38.2%
	(5)	(20)	(9)	(34)
Total	32.6%	53.9%	13.5%	100%
	(29)	(48)	(12)	(89)

Data presented as percentages (counts); asterisks indicate high-caseload sonographers.

Seventy-six respondents participated in the assessment of diagnostic accuracy. Twenty-eight of these (35.5%) assessed LUS to be ‘always reliable’ in ruling *out* pneumothorax; another 41 (53.9%) felt that LUS was ‘frequently reliable’. In contrast, 16 respondents (21.3%) assessed LUS to be ‘always reliable’ in ruling *in* pneumothorax; a further 52 (68.6%) felt that it was ‘frequently reliable’.

For the purpose of subgroup analysis, respondents were assigned to either a ‘low-caseload group’ or a ‘high-caseload group’, depending on the frequency of exposure to suspected pneumothorax cases and the frequency of LUS usage. For example, physicians who are involved in the management of more than one suspected pneumothorax case per week and who frequently use ultrasound in this scenario can be expected to perform 50 to 100 examinations per year. Forty respondents (44.9%) were assigned to the high-caseload group (Table 1).

**Figure 1:**
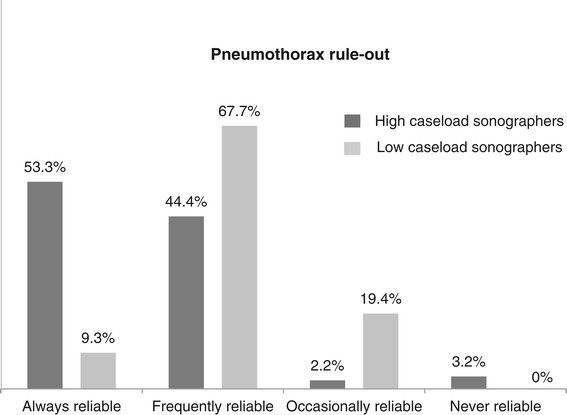
**Assessment of accuracy of LUS to rule out pneumothorax.** Respondents grouped as either high-caseload sonographers or low-caseload sonographers (*n* = 76).

**Figure 2:**
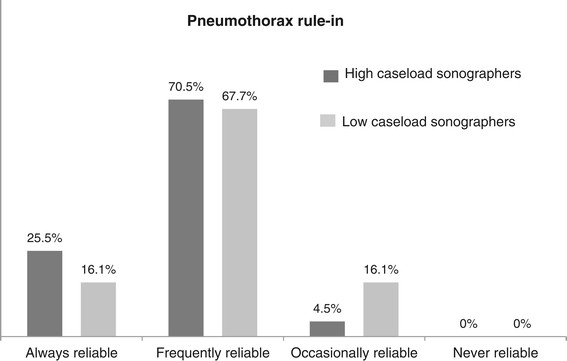
**Assessment of accuracy of LUS to rule in pneumothorax.** Respondents grouped as either high-caseload sonographers or low-caseload sonographers (*n* = 76).

The proportion of physicians who felt that LUS was ‘always reliable’ in ruling out pneumothorax was significantly higher in the high-caseload group (*p* = 0.0003) (Figures 1 and 2).

**Figure 3:**
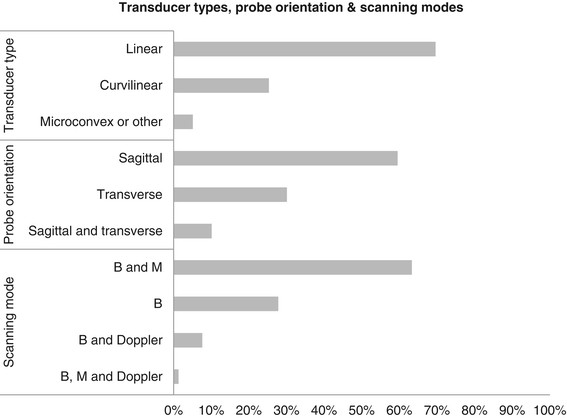
**Selection of transducers, probe orientations and scanning modes (*n*** **= 78).**

Figure 3 illustrates respondents' preferences regarding selection of transducers, probe orientation and scanning modes. In total, 16 different combinations of transducer types, probe orientations and scanning modes were reported (Table 2). There were no statistically significant differences regarding transducer selection and probe orientation between high-caseload sonographers and low-caseload sonographers. However high-caseload sonographers used M-mode scanning in addition to B-mode scanning more frequently (*p* = 0.019).

**Table 2:** **Frequently reported combinations of transducer types, probe orientations and scanning modes (*n*** **= 78)**

**Transducer type**	**Probe orientation**	**Scanning mode**	**Reported usage**
Linear	Sagittal	B-mode and M-mode	30.8%
			(24)
Linear	Transverse	B-mode and M-mode	12.8%
			(10)
Linear	Sagittal	B-mode	10.2%
			(8)
Curvilinear	Sagittal	B-mode and M-mode	7.6%
			(6)
Curvilinear	Transverse	B-mode	6.4%
			(5)
Curvilinear	Sagittal	B-mode	5.1%
			(4)
Linear	Sagittal and transverse	B-mode and M-mode	5.1%
			(4)
Linear	Transverse	B-mode and Doppler-mode	3.8%
			(3)
Linear	Transverse	B-mode	3.8%
			(3)
Curvilinear	Transverse	B-mode and M-mode	2.6%
			(2)
Other combinations			11.5%
			(9)

Data presented as percentages (counts).

Seventy-eight LUS users selected at least one mode of documentation. Thirty-two of these (41.0%) used a combination of two modes of documentation, whereas 22 respondents (28.2%) used a combination of three modes or more. The most frequently selected combination was storage of a video clip on the ultrasound machine along with a written entry in the patient's notes (Table 3).

**Table 3:** **Frequently reported combinations of image storage and documentation of lung ultrasonography (*n*** **= 78)**

**Mode of storage and documentation**	**Reported usage**
Storage of video clip on U/S machine and entry in patients' notes	30.8%
	(24)
Printout of still image and storage of video clip on U/S machine and entry in patients notes	19.2%
	(15)
Transfer of exam into PACS	12.8%
	(10)
Storage of video clip on U/S machine	10.3%
	(8)
Printout of still image and entry in patients' notes	10.3%
	(8)
Entry in patients' notes	6.4%
	(5)
Various other modes of storage and documentation	10.3%
	(8)

*U/S* ultrasound, *PACS* picture archiving and communication system. Data presented as percentages (counts).

Eighty respondents (89.9%) were actively involved in teaching LUS for the diagnosis of pneumothorax to medical and non-medical staff.

## Discussion

Our survey revealed significant variations both in exposure to patients with suspected pneumothorax and the use of LUS. We estimate that a typical respondent to our survey uses LUS for the diagnosis of suspected pneumothorax approximately once a week, corresponding to an average of 50 examinations per year. To us the correlation between frequency of exposure to pneumothorax cases and the inclination to use LUS suggests that clinicians who have had a more intense experience of the enhanced diagnostic capability offered by point-of-care lung ultrasound are more avid in the uptake of LUS.

Physicians' perception of diagnostic accuracy was present but not complete. High-caseload sonographers were particularly confident to rule out pneumothorax. A possible explanation is that these clinicians have had a learning experience enabling them to appreciate high specificity of LUS while acknowledging that sensitivity of LUS remains limited, particularly in difficult scenarios, such as lung emphysema or severe asthma. If our interpretation was correct, it would corroborate the recently published meta-analysis by Alrajab and co-workers [6].

We were not surprised to find a large variation of combinations of transducers, probe orientations and ultrasound modes reported by respondents. A review of published clinical studies and recommendations suggests that the choice of specific technique for LUS is relevant but this has not been systematically evaluated [2],[4],[6]. Few investigators specifically addressed the issue of differences between transducers regarding the capability to detect lung sliding. Targhetta et al. noted that 7.5 MHz linear transducers facilitated the detection of lung sliding [7]. Lichtenstein and Menu advised against the use of 2.75 MHz echocardiography transducers due to the lower sensitivity of these transducers as compared to microconvex transducers operating at higher frequencies [8]. Soldati et al., while using convex transducers in their study of occult pneumothorax, recommended the use of high-frequency linear probes in difficult cases, e.g. when lung sliding is reduced [9]. The majority of respondents to our survey preferred linear transducers. This is arguably a very reasonable choice, given that there is some indication that these types of transducers offer better sensitivity for the detection of lung sliding.

The use of B-mode scanning for pneumothorax seems intuitively reasonable; it is the only mode available on every ultrasound machine and usually serves as a starting point for any ultrasound exam. Lung sliding can also be visualized on M-mode and Doppler-mode. Whether one mode is superior over the others or if the combined use of several modes enhances diagnostic accuracy has not been investigated so far. The majority of respondents to our survey combined B-mode and M-mode scanning. There are several approaches to interpreting this finding. One interpretation is that B-mode is used as the primary scanning mode, and M-Mode is added to confirm the initial diagnostic findings. Another interpretation is that both scanning modes are used in a complementary way in order to reduce the risk of an equivocal result. Finally M-mode may be perceived as particularly useful to document the results of the examination as a printout of a still image.

Adequate documentation of the results of lung ultrasound is cumbersome. LUS is different from any other type of sonography as visualisation of the shape and structure of the organ in question is of little relevance. By contrast, the dynamic appearance and disappearance of acoustic artefacts caused by movements of the visceral pleura in relation to the parietal pleura is visualised and interpreted during the examination. Printouts of still images are of unproven value when it comes to reproducing or presenting the result of the exam. Storage of video clips on the ultrasound machine's hard disk is easily feasible and enables reviewing of the exam but does not meet the criteria of good medical practice [10]. Writing an entry in the patient's notes will both confirm that the exam has taken place and what the sonographer's interpretation of the findings was. Again, there is no way of reproducing the result. Data transfer into a picture archiving and communication system (PACS) is recommended but this requires elaborate IT structure and may therefore not be available to many sonographers [11]. The fact that participants of our survey reported multiple modalities of documentation may reflect the absence of one simple and reliable way of documentation.

The fact that more than nearly 90% of respondents of our survey were involved in teaching of LUS leads us to believe that physicians who participated in this survey share a credo that LUS is a useful clinical tool that should be made available to colleagues and co-workers.

To our knowledge, this survey is the first report on the use of LUS in the diagnosis of pneumothorax in routine clinical practice. The diagnostic potential of LUS for the confirmation or the exclusion of pneumothorax has been demonstrated in numerous studies. However, the vast majority of these studies were performed in selected clinical scenarios [6]. Until now, it has not been known to what extent LUS is used by physicians involved in the management of patients with suspected pneumothorax in unselected, routine clinical settings and how it is valued.

Our study has its limitations. The questionnaire was brief and participants were asked to give their assessments in a categorical fashion. We were aware of the fact that more detailed information might have been useful. However, we felt that a more time-consuming survey participation would have resulted in a lower response rate. We focused on asking questions on ‘what’ sonographers did and not on ‘why’ they did it, as the latter would have required us asking open questions. Those types of questions are difficult to analyse and interpret [12]. Our study may also be criticized for what might be considered a low response rate (26%) suggesting a degree of self-selection of participants. It is conceivable that more experienced or enthusiastic users of LUS were more inclined to respond. The fact that a very high proportion of participants reported involvement in teaching and training supports this notion. On all accounts, users of point-of-care ultrasonography are a very diverse and heterogeneous group of physicians and there may just not be *the* representative sample as such. Moreover, the main purpose of the study was to gather information on the degree of diversity of current practice and not to assess adherence to a guideline or implementation of recommendations issued by a professional body or regulatory authority.

## Conclusions

A proportion of physicians who are actively involved in the management of patients with suspected pneumothorax use lung ultrasound in the diagnostic workup, albeit to a varying extent. Confidence to rule out pneumothorax by LUS is present, but is far from comprehensive. Frequent exposure to patients with suspected pneumothorax is associated both with more frequent usage of LUS and higher confidence to make a diagnosis based on LUS findings. Considerable variations exist in the technical performance of LUS. We conclude that further research of the use of ultrasound in the diagnosis of pneumothorax is required. Physicians will benefit from firm knowledge regarding the best choice of transducers, probe orientation and scanning modes in order to be able to achieve the highest possible diagnostic accuracy and reliable documentation of examination results without excessive use of time and resources.

## Competing interests

The authors declare that they have no competing interests.

## Authors’ contributions

TB conceived and designed the study, contributed to the acquisition, analysis and interpretation of the data, drafting of the manuscript, and takes responsibility for the integrity of the data and the accuracy of the data analysis. TF and TM contributed to the study conception, analysis and interpretation of the data, drafting of the manuscript and critical revisions for important intellectual content. All authors read and approved the final manuscript.

## Additional file

## Supplementary Material

Additional file 1: **English language version of the survey questionnaire.** Questions pertained to frequency of involvement in the management of patients with suspected pneumothorax, frequency of LUS use in this setting, preferences regarding technical aspects of LUS use, physicians' perception of diagnostic accuracy and involvement in teaching of LUS. Click here for file
