# Supplementary material for: Current practice of lung ultrasonography (LUS) in the diagnosis of pneumothorax: a survey of physician sonographers in Germany
Source: Crit Ultrasound J. 2014 Oct 15;6(1):16. doi: 10.1186/s13089-014-0016-y (PMC4233327; doi:10.1186/s13089-014-0016-y)
Supplement: Additional file 1: — English language version of the survey questionnaire. Questions pertained to frequency of involvement in the management of patients with suspected pneumothorax, frequency of LUS use in this setting, preferences regarding technical aspects of LUS use, physicians' perception of diagnostic accuracy and involvement in teaching of LUS. [file s13089-014-0016-y-S1.docx]

Background: The purpose of this study was to survey the current practice of the use of lung ultrasonography (LUS) in the diagnosis of pneumothorax.

Methods: Physician sonographers, accredited for diagnostic ultrasonography in surgery, anaesthesia, and medicine were studied. Questions addressed the frequency of exposure to patients with suspected pneumothorax, frequency of LUS-use, preferences regarding technical aspects of LUS examination, assessment of diagnostic accuracy of LUS and involvement in teaching.

Results: 55.1 % of respondents used LUS “always” or “frequently” for suspected pneumothorax. 35.5% of physicians rated LUS as “always reliable” in ruling out pneumothorax, and 21.3% of respondents rated LUS as “always reliable” in ruling in pneumothorax. The mode of performing LUS for pneumothorax was highly variable.

Statistically significant differences where found regarding the likelihood of LUS-usage, the combined use of M-Mode and B-mode scanning, and the confidence to exclude pneumothorax based on LUS findings for physicians with frequent exposure to pneumothorax cases.

Conclusions: Physicians’ use of LUS in the diagnosis of pneumothorax is modest. Confidence in diagnostic accuracy is not comprehensive. Further research is required to establish the most efficient way of performing LUS in this scenario to achieve highest possible diagnostic accuracy and reliable documentation of examination results.

Key words: ultrasonography, pneumothorax, technology assessment, questionnaire
